# Association between BMI and age at menarche or spermarche among both sexes: Findings from six successive national surveys in China

**DOI:** 10.7189/jogh.14.04099

**Published:** 2024-05-10

**Authors:** Shi Di, Ma Ning, Liu Yunfei, Dang Jiajia, Zhong Panliang, Cai Shan, Chen Ziyue, Ma Jun, Song Yi

**Affiliations:** 1 Institute of Child and Adolescent Health, School of Public Health, Peking University, Beijing, China; 2 National Health Commission Key Laboratory of Reproductive Health, Peking University, Beijing, China; 3 Vanke School of Public Health, Tsinghua University, Beijing, China

## Abstract

**Background:**

To explore trends of the association between body mass index (BMI) and age at menarche or spermarche and its urban-rural disparities from 1995 to 2019.

**Methods:**

A total of 912 753 children and adolescents – including 519 940 9–18 years old girls and 392 813 11–18 years old boys – were involved in six successive cross-sectional surveys conducted across 30 provinces in China from 1995 to 2019. Data on menarche and spermarche was collected using the status quo method, where same-gender physicians conducted face-to-face interviews to determine if children and adolescents had experienced their first menstrual cycle or ejaculation (yes/no). The median age at menarche or spermarche was estimated by probit analysis. Anthropometric measurements measured the height and weight of the study subjects. Children and adolescents were classified into thinness, normal range of weight, overweight, and obesity. *t* test was used to compare the differences in BMI between premenarchal and postmenarchal girls or prespermarcheal and postspermarcheal boys. Logistic regression was used to explore the associations between BMI/nutritional status and menarche or spermarche stratified by urban or rural residency status.

**Results:**

From 1995 to 2019, BMI in all age groups growth over time, and the values of BMI among children and adolescents under 15 who had menarche or spermarche were more significant than those without menarche or spermarche. In 2019, for girls, thinness was associated with delayed menarche (odds ratio (OR) = 0.26; 95% confidence interval (CI) = 0.24–0.28), while overweight (OR = 1.99; 95% CI = 1.85–2.14) and obesity (OR = 2.20; 95% CI = 1.92–2.53) was associated with advanced menarche. For boys, thinness was associated with delayed spermarche (OR = 0.71; 95% CI = 0.65–0.78), overweight was associated with advanced spermarche (OR = 1.08; 95% CI = 1.01–1.15) while obesity had no association with spermarche. The OR between BMI and menarche in 1995 was 1.35 (95% CI = 1.33–1.37), which decreased to 1.19 (95% CI = 1.18–1.20) by 2019. The OR between BMI and spermarche in 1995 was 1.10 (95% CI = 1.09–1.11), which decreased to 1.02 (95% CI = 1.02–1.03) by 2019. The trends by urban-rural stratification were consistent with the total sample.

**Conclusions:**

We have established a dose-response relationship between BMI and menarche in girls, whereas the association appears to be nonlinear in boys, and the associations were diminishing. Similar findings were observed in both urban and rural areas. Considering the dual adverse effects of obesity and early puberty on health, the results of this study suggest that sexual health education should be strengthened, especially among obese girls. Further research on the influencing factors and biological mechanisms of early puberty will be beneficial.

Pubertal development influences not only the current health of children and adolescents, but also their physical and mental health in adulthood, and the health of the next generation [1]. Many countries have discovered that the puberty occurred earlier and earlier in both sexes [2], especially for girls, with disparities between urban and rural areas. Early puberty was associated with numerous adverse health outcomes [3]. Menarche was typically considered an essential indicator of female puberty. Moreover, there were numerous indicators of male puberty, among which the age at spermarche was important [4]. Although genetic and environmental factors usually impact puberty development [5,6], the trends of earlier age at menarche or spermarche might not be driven by genetic consideration during the short term for a few decades. Globally, along with the secular early trend in age at menarche or spermarche, the prevalence of overweight and obesity among children and adolescents has rapidly increased in past decades. In China, the prevalence of overweight and obesity in children and adolescents increased from 5.3% in 1995 to 24.2% in 2019 [7,8]. As for environmental factors, especially nutritional status, were more crucial for the early puberty.

In girls, numerous studies have confirmed the association between higher or excessively growing BMI levels and early menarche, puberty growth spurt, and age at peak height velocity [9,10]. The conclusions regarding the relationship between BMI and puberty in boys were inconsistent due to variations in index and samples [11,12]. However, most previous studies have used single cross-sectional, unrepresentative data or clinical intervention studies to explore the association of BMI with puberty in single-sex, and the results needed to be updated. There needed to be more studies conducted whether the associations between BMI and age at menarche or spermarche in both sexes have changed over time and whether there have been urban-rural disparities using multiple continuous data at the national level with high comparability on the same life stage.

Chinese National Surveys on Students' Constitution and Health (CNSSCH) was a nationwide student health survey conducted every five years in China, including of Han children and adolescents aged seven to 18 in 30 provinces, municipalities, and autonomous regions to collect basic information regarding their height, weight, menarche, and spermarche [13]. This study utilised the CNSSCH data set from 1995 to 2019 to explore the association trends between BMI and age at menarche or spermarche in both sexes and the urban-rural disparities. We hypothesised that the association between BMI and age at menarche and spermarche diminished over time and that there are differences between sexes in urban and rural areas. This study aimed to investigate trends of the association between BMI and age at menarche or spermarche in both sexes and its urban-rural disparities using constantly comparable large population data.

## METHODS

### Subject

**Figure 1:**
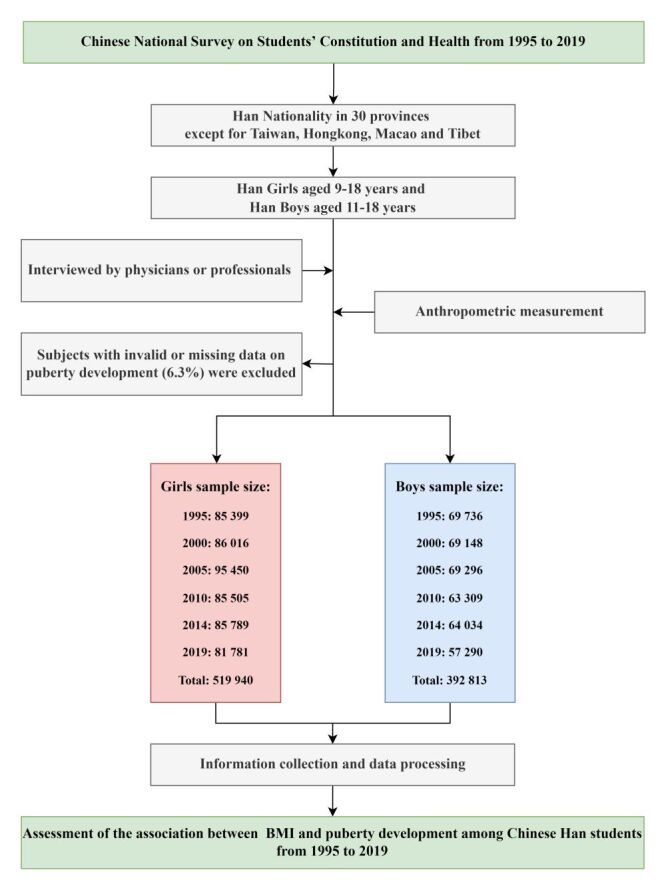
Flowchart of Chinese National Surveys on Students' Constitution and Health and analysis.

We investigated the Han sample from 30 Chinese provinces in 1995, 2000, 2005, 2010, 2014 and 2019 CNSSCHs, a total of 912 753 children and adolescents – including 519 940 9–18 years old girls and 392 813 11–18 years old boys – were involved. The survey procedure was the same at all CNSSCH time points to ensure the comparability and reliability of the data [13]. In brief, all children and adolescents were recruited by stratified cluster sampling; that is, sampling took place in classes selected randomly from each grade in the selected schools. Sampling yielded equal numbers at three socioeconomic status groups (i.e. upper, moderate, low) at the regional level defined by regional gross domestic product, total yearly income per capita, average food consumption per capita, natural growth rate of population, and the regional social welfare index, based on National Bureau of Statistics of the People's Republic of China published data. The schools (urban and rural) in every socioeconomic status group of each province were randomly selected in 1985. Then, the schools that carried out the survey remained the same in each survey (**Figure 1**). The ratio of urban to rural in each survey was around 1:1. The sample size were presented in Table S1 in the **Online Supplementary Document**.

### Measures

Individual data on menarche or spermarche were acquired by the status quo method through surveys. In specific, face-to-face interviews were conducted with children and adolescents by physicians of the same gender. Children and adolescents were asked if they had ever experienced their first menarche or their spermarche in the past. During the interviews, the girls were asked whether they had started menstruating and the boys were asked whether they had experienced their first ejaculation. Their responses were recorded as ‘yes’ if they indicated having begun menstruation/spermarche or ‘no’ if they reported not having started yet. Given that children and adolescents had received relevant health education in class, they could understand their physiological situation and recall whether it had already happened. The physicians underwent thorough training to ensure that they could accurately explain menstruation to young children and adolescents. Children and adolescents were classified into four categories: thinness, normal range of weight, overweight, and obesity, according to BMI by sex-age-specific references recommended by the International Obesity Task Force (IOTF). Children and adolescents who lacked height and weight information, did not understand menarche or spermarche after explanation, could not remember or refused to answer questions were regarded as invalid cases and excluded from the study (6.3%).

### Statistical analysis

Continuous variables were represented as mean (standard deviation). Categorical variables were shown as numbers (percentages). *t* test was used to compare the differences in BMI between premenarchal and postmenarchal girls or prespermarcheal and postspermarcheal boys. Probit analysis was employed to estimate the age and the 95% confidence interval (CI) at menarche or spermarche for all children and adolescents, including the 10th, 25th, 50th (median), 75th, and 90th percentiles. The ages were recorded and calculated in decimals, such as 8.00–8.99 years and 9.00–9.99 years. Age at menarche was estimated when the proportion of children and adolescents who had reached menarche or spermarche was equal to 10, 25, 50, 75, and 90%. A cumulative standard curve was fit to the proportion of children and adolescents of each age who reached menarche or spermarche, and the median age at menarche or spermarche was the corresponding age at which 50% of children and adolescents in the population could be predicted to have reached menarche or spermarche.

Logistic regression was used to model the association between BMI (continuous variable) and menarche or spermarche, and the association between nutritional status (categorical variable) and menarche or spermarche in both sexes, adjusted for age, province, urban or rural residency status, and socioeconomic status. The standard weight group was considered as the reference group. In addition, all study subjects were included in the Logistic regression equation, using BMI and survey year as independent variables to test whether the interaction term of BMI and survey year were statistically significant to determine whether the association of BMI and menarche or spermarche changed with survey year. A linear trend test was conducted using the nutritional status variable as a continuous variable by assigning the median values of each group to evaluate the presence of a dose-response relationship. The percent relative standard error (PRSE) test was performed to test the reliability of regression coefficients. In this test, the PRSE is calculated as a percentage by dividing the standard error of the coefficient estimate by the magnitude of the estimate itself. This value is then multiplied by 100 to obtain the percentage. PRSE<25% could be considered as a reliable estimation; it suggests that the coefficient estimate is stable and has low sampling variability. Besides, the interaction analysis was used to test the differences between groups in urban or rural residency status. We classified the study subjects as thinness, normal range of weight, overweight, and obesity according to World Health Organization (WHO) references, as a sensitivity analysis. The significance level was accepted at two-tailed *P* < 0.05. All analyses were conducted with SPSS, version 26.0 (IBM, Armonk, New York) and GraphPad Prism 8.0.2 (GraphPad Software, Inc).

## RESULTS

### Essential characters of BMI and age at menarche or spermarche in both sexes

**Figure 2:**
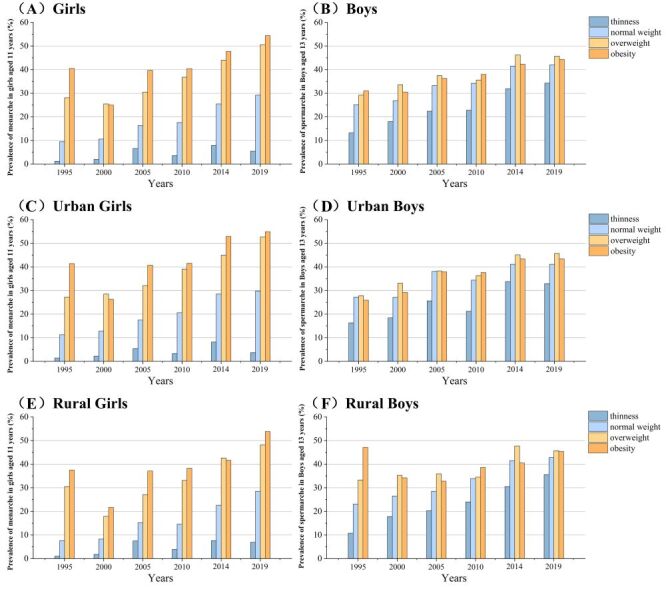
Prevalence of girls aged 11 years who reached menarche and boys aged 13 years who reached spermarche from 1995 to 2019 by nutritional status among different areas.

**Figure 3:**
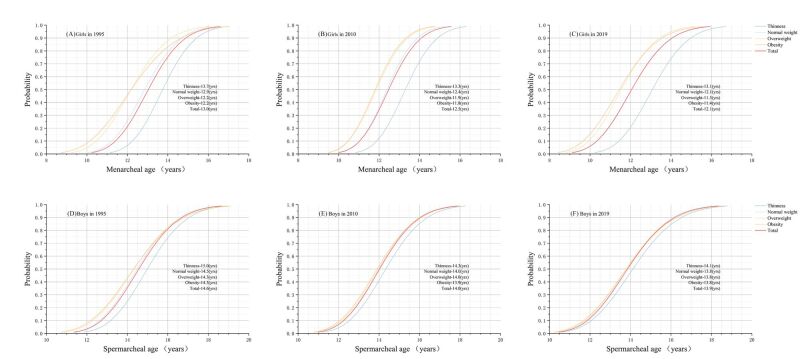
Probit plots of age at menarche or spermarche by nutritional status in 1995, 2010 and 2019.

There was no significant difference in urban or rural resident status between the primary and final samples, and the average age of children and adolescents in the final sample was slightly higher than in the primary sample (Table S2 in the **Online Supplementary Document**). From 1995 to 2019, BMI in all age groups increased over time in both sexes, and the BMI of children and adolescents under 15 who had menarche or spermarche was greater than that of children and adolescents without menarche or spermarche, the results of *t* test were significant in each age subgroup (**Table 1**). The prevalence of menarche in 11-year-old girls and spermarche in 13-year-old boys both increased from 1995 to 2019. The prevalence of menarche or spermarche was lower among thin children and adolescents than among normal range of weight children and adolescents. In contrast, it was higher among overweight and obese children and adolescents. In girls, the prevalence of menarche was lower among those who were overweight compared to those who were obese (**Figure 2**, panel A). In boys, the prevalence of spermarche was higher among those who were overweight (**Figure 2**, panel B). Among girls, the gap of menarche in different nutritional status groups was more pronounced, and this disparity exists similarly between urban and rural areas (**Figure 2**, panels C–F). The median age at menarche or spermarche was larger in the thinness group and smaller in the overweight and obese groups than the normal range of weight groups. The difference in the median age at spermarche between the thin and obese groups decreased from 0.7 years in 1995 to 0.3 years in 2019 (**Figure 3**, panels A–F). The median ages at menarche or spermarche of different nutritional status in urban children and adolescents and rural peers from 1995 to 2019 were presented in Figures S1–S6 in the **Online Supplementary Document**.

**Table 1:** Mean (SD) BMI in premenarcheal and postmenarcheal girls aged 9–18 y and in prespermarcheal and postspermarcheal boys aged 11–18 y from 1995 to 2019

Ages (y)	1995			2000			2005		
	**Premenarcheal**	**Postmenarcheal**	***P*-value**	**Premenarcheal**	**Postmenarcheal**	***P*-value**	**Premenarcheal**	**Postmenarcheal**	***P*-value**
9	15.1 (1.8)	16.4 (3.9)	0.006	15.6 (2.2)	16.5 (1.8)	<0.001	15.9 (2.3)	15.4 (1.9)	<0.001
10	15.6 (1.9)	17.8 (2.9)	<0.001	16.0 (2.3)	17.8 (2.8)	<0.001	16.4 (2.5)	16.9 (2.8)	<0.001
11	16.1 (2.1)	18.4 (2.6)	<0.001	16.5 (2.4)	18.6 (2.8)	<0.001	16.9 (2.6)	18.5 (3.1)	<0.001
12	16.5 (2.1)	18.3 (2.4)	<0.001	16.7 (2.2)	18.6 (2.7)	<0.001	17.1 (2.6)	18.8 (2.8)	<0.001
13	17.0 (2.1)	18.6 (2.3)	<0.001	17.0 (2.4)	18.7 (2.6)	<0.001	17.2 (2.3)	19.0 (2.8)	<0.001
14	17.5 (2.3)	18.9 (2.3)	<0.001	17.5 (2.3)	19.1 (2.6)	<0.001	17.6 (2.4)	19.4 (2.8)	<0.001
15	18.9 (2.6)	19.4 (2.3)	<0.001	18.6 (2.5)	19.6 (2.5)	<0.001	18.9 (2.6)	19.8 (2.7)	<0.001
16–18	20.1 (2.3)	20.1 (2.2)	0.890	20.2 (2.2)	20.3 (2.4)	0.572	20.4 (2.6)	20.2 (2.5)	0.284
**Ages (y)**	**2010**			**2014**			**2019**		
	**Premenarcheal**	**Postmenarcheal**	***P*-value**	**Premenarcheal**	**Postmenarcheal**	***P*-value**	**Premenarcheal**	**Postmenarcheal**	***P*-value**
9	16.2 (2.4)	17.4 (2.8)	<0.001	16.7 (2.7)	18.0 (3.1)	<0.001	16.9 (2.9)	18.5 (3.5)	<0.001
10	16.8 (2.6)	18.9 (3.2)	<0.001	17.2 (2.9)	18.6 (3.1)	<0.001	17.5 (3.1)	19.3 (3.6)	<0.001
11	17.1 (2.7)	19.3 (2.9)	<0.001	17.5 (2.9)	19.5 (3.1)	<0.001	17.8 (3.0)	19.9 (3.2)	<0.001
12	17.2 (2.5)	19.2 (2.9)	<0.001	17.6 (2.8)	19.5 (3.1)	<0.001	17.8 (3.1)	20.0 (3.4)	<0.001
13	17.4 (2.6)	19.3 (2.9)	<0.001	17.5 (2.7)	19.7 (3.0)	<0.001	18.1 (2.9)	20.2 (3.4)	<0.001
14	17.6 (2.4)	19.6 (2.8)	<0.001	18.2 (3.0)	20.0 (3.0)	<0.001	20.0 (3.9)	20.7 (3.4)	0.001
15	18.2 (2.9)	19.9 (2.7)	<0.001	18.9 (2.6)	20.3 (2.9)	0.001	21.0 (3.6)	21.0 (3.2)	0.927
16–18	19.7 (3.0)	20.3 (2.5)	0.021	20.6 (2.1)	20.7 (2.8)	0.860	21.3 (3.1)	21.2 (3.2)	0.358
**Ages (y)**	**1995**			**2000**			**2005**		
	**Prespermarcheal**	**Postspermarcheal**	***P-*value**	**Prespermarcheal**	**Postspermarcheal**	***P-*value**	**Prespermarcheal**	**Postspermarcheal**	***P-*value**
11	16.5 (2.3)	17.6 (2.9)	<0.001	17.1 (2.8)	17.8 (2.9)	<0.001	17.7 (3.1)	18.2 (3.4)	0.006
12	16.9 (2.3)	18.1 (2.7)	<0.001	17.5 (2.9)	18.1 (2.8)	<0.001	18.0 (3.2)	18.8 (3.5)	<0.001
13	17.6 (2.4)	18.3 (2.5)	<0.001	17.9 (2.8)	18.6 (3.0)	<0.001	18.3 (3.2)	19.0 (3.2)	<0.001
14	18.0 (2.4)	18.7 (2.5)	<0.001	18.4 (2.8)	18.9 (2.9)	<0.001	18.6 (3.1)	19.3 (3.2)	<0.001
15	18.7 (2.5)	19.0 (2.3)	<0.001	19.0 (2.9)	19.4 (2.8)	<0.001	19.1 (3.0)	19.6 (3.1)	<0.001
16-18	19.7 (2.4)	19.9 (2.2)	0.001	20.0 (2.9)	20.3 (2.8)	<0.001	20.1 (3.3)	20.3 (3.0)	0.003
**Ages (y)**	**2010**			**2014**			**2019**		
	**Prespermarcheal**	**Postspermarcheal**	***P*-value**	**Prespermarcheal**	**Postspermarcheal**	***P-*value**	**Prespermarcheal**	**Postspermarcheal**	***P-*value**
11	18.4 (3.4)	18.8 (3.5)	0.116	18.9 (3.6)	19.1 (3.7)	0.217	19.3 (3.9)	19.8 (4.3)	0.020
12	18.7 (3.5)	19.5 (3.8)	<0.001	19.2 (3.5)	19.9 (3.7)	<0.001	19.7 (3.9)	20.4 (4.1)	<0.001
13	19.0 (3.4)	19.5 (3.3)	<0.001	19.6 (3.7)	20.0 (3.6)	<0.001	20.0 (4.0)	20.5 (4.1)	<0.001
14	19.3 (3.3)	19.7 (3.3)	<0.001	19.7 (3.6)	20.3 (3.6)	<0.001	20.5 (4.1)	20.9 (4.0)	0.001
15	19.8 (3.5)	20.1 (3.3)	0.008	20.1 (3.4)	20.6 (3.5)	0.001	20.9 (4.1)	21.1 (3.8)	0.117
16–18	20.4 (3.4)	20.6 (3.1)	0.080	21.0 (3.6)	21.2 (3.5)	0.126	22.1 (4.1)	21.8 (3.9)	0.029

BMI – body mass index, SD – standard deviation

### Association between BMI and menarche or spermarche in both sexes

**Figure 4:**
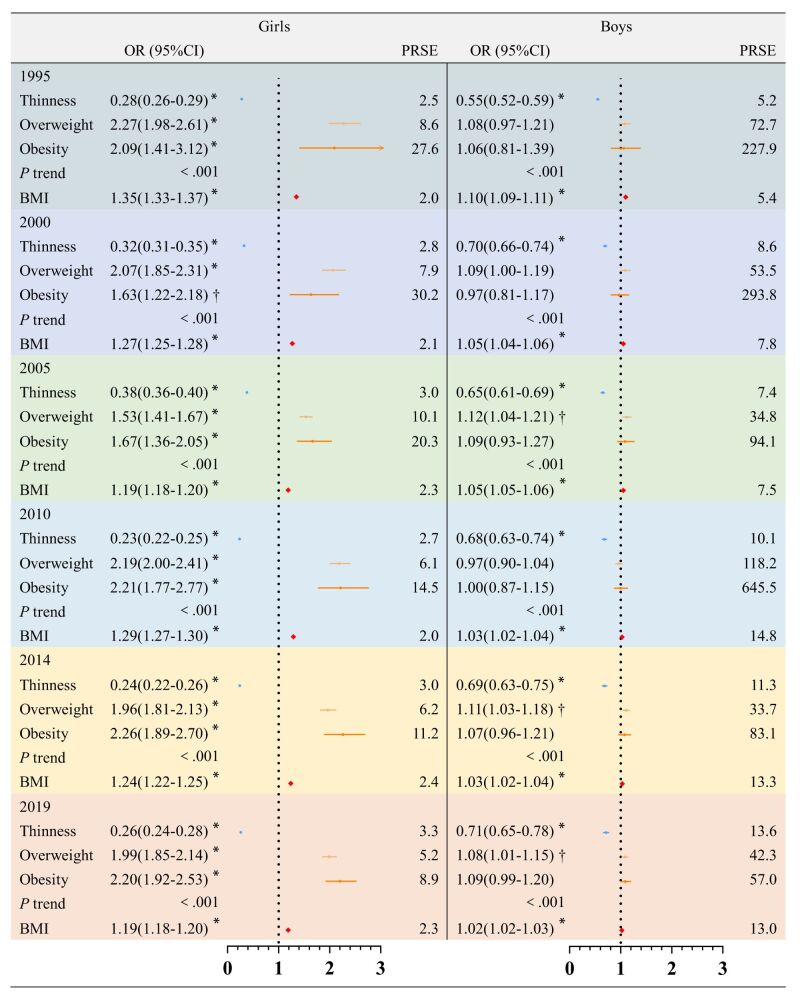
Association between nutritional status and menarche or spermarche from 1995 to 2019. Adjusted for age, province, socioeconomic status and urban or rural residency status. *P < 0.001. †*P* < 0.05. BMI – body mass index, CI – confidence interval, OR – odds ratio, PRSE – percent relative standard error

**Figure 5:**
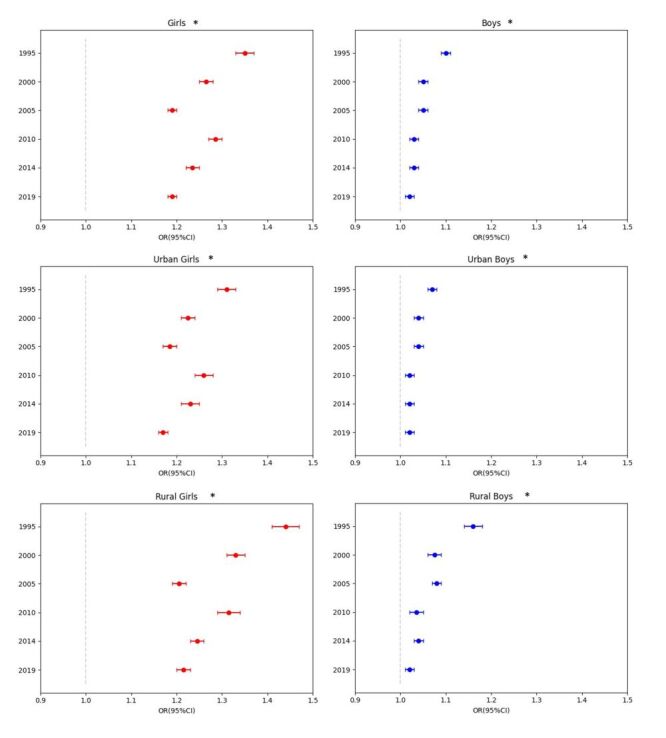
Association between BMI and menarche or spermarche from 1995 to 2019. Adjusted for age, province, socioeconomic status and urban or rural residency status. *The interaction of BMI and survey year was significant (*P* < 0.001). BMI – body mass index

The associations between nutritional status and menarche or spermarche in both sexes are shown in **Figure 4**. In contrast, the associations between BMI and menarche or spermarche in both sexes are shown in **Figure 5**. After adjusting for confounding factors, in 9–18 years old girls in 2019, thinness was related to delayed menarche (odds ratio (OR) = 0.26; 95% CI = 0.24–0.28), overweight (OR = 1.99; 95% CI = 1.85–2.14) and obesity were related to advanced menarche (OR = 2.20; 95% CI = 1.92–2.53). In 11–18 years old boys, thinness was related to delayed spermarche (OR = 0.71; 95% CI = 0.65–0.78), overweight was related to advanced spermarche (OR = 1.08; 95% CI = 1.01–1.15) and obesity has no association with spermarche. The results of the linear trend tests were all statistically significant (**Figure 4**). The strength of the association between BMI and menarche has shown a significant decline over the past 24 years (β_SE_ = -0.025(0.001), *P*_interaction_<0.001). The OR between BMI and menarche in 1995 was 1.35 (95% CI = 1.33–1.37), which decreased to 1.19 (95% CI = 1.18–1.20) by 2019. This indicates that from 1995 to 2019, for each unit increase in BMI, the probability of menarche decreased from a 35% increase to a 19% increase. In boys, the association between BMI and spermarche also showed a significant decline (β_SE_ = -0.003(<0.001), *P*_interaction_<0.001), with the OR dropping from 1.10 (95% CI = 1.09–1.11) in 1995 to 1.02 (95% CI = 1.02–1.03) by 2019. This means that the increase in the probability of spermarche per unit increase in BMI has reduced from 10 to 2% over the same period. PRSE tests showed a reliable estimation. There was no difference between the sensitivity analysis results and the primary results (Figure S7 in the **Online Supplementary Document**).

### The urban-rural disparity in the association between BMI and menarche or spermarche in both sexes

The associations between nutritional status and menarche or spermarche were approximate the same by urban or rural residency status in both sexes (Figures S8–S9 in the **Online Supplementary Document**). In urban girls, the association between BMI and menarche in 2019 (OR = 1.17; 95% CI = 1.16–1.18) was lower than the association in 1995 (OR = 1.31; 95% CI = 1.29–1.33), the interaction of BMI and survey year was significant (β_SE_ = -0.002(<0.001), *P*_interaction_<0.001). In urban boys, the association between BMI and spermarche in 2019 (OR = 1.02; 95% CI = 1.01–1.03) was lower than the association in 1995 (OR = 1.07; 95% CI = 1.06–1.08), the interaction of BMI and survey year was significant (β_SE_ = -0.002(<0.001), *P*_interaction_<0.001). In rural girls, the association between BMI and menarche in 2019 (OR = 1.22; 95% CI = 1.20–1.23) was lower than the association in 1995 (OR = 1.44; 95% CI = 1.41–1.47), the interaction of BMI and survey year was significant(β_SE_ = -0.004(<0.001), *P*_interaction_<0.001). In rural boys, the association between BMI and spermarche in 2019 (OR = 1.02; 95% CI = 1.02–1.03) was lower than the association in 1995 (OR = 1.16; 95% CI = 1.14–1.18), the interaction of BMI and survey year was significant(β_SE_ = -0.004(<0.001), *P*_interaction_<0.001).

## DISCUSSION

The results indicated that the association between BMI and age at menarche or spermarche significantly reduced over time in urban and rural areas. We have established a dose-response relationship between nutritional status and menarche in girls. The association between nutritional status and spermarche appears to be nonlinear relationship in boys.

In the previously published study of our research team, we found a significant advance trend in the median age of menarche in Chinese Han girls and the median age of spermarhce in Chinese Han boys in the past 25 years [14,15]. Previous studies have showed a similar phenomenon [12,16]. Early puberty might result in negative influence on health consequences such as decreased quality of life [17], increased health risk behaviours [18], and an elevated risk of cancer [19]. To prevent and intervene, it is essential to investigate the influencing factors and underlying mechanisms of puberty development. Our findings showed that thinness was associated with delayed menarche or spermarche; overweight and obesity were associated with advanced menarche in girls, overweight was associated with advanced spermarche in boys [2].

This study provided evidence that BMI may be associated with puberty in both sexes. Studies showed that BMI was related to earlier pubertal maturation in girls, similar to our results [20,21]. The potential biological mechanism relating BMI with earlier onset of menarche in girls may involve the regulatory action of leptin. Estrogen may promote the secretion of leptin by adipose cells [22,23]. Studies indicated that for each increase of one ng/ml in serum leptin, the age at menarche was correlated with an advancement by one month, and an increase of one kg in body fat was associated with menarche occurring 13 days earlier [24,25]. As a signal for the GnRH (gonadotropin-releasing hormone) pulse generator in the hypothalamus, leptin induced the onset of puberty [26,27]. Additionally, the receptors of other adipokines, such as adiponectin, may also contribute to the earlier onset of pubertal development; adiponectin concentrations in obese children were lower than in children of normal range of weight, and adiponectin may inhibit GnRH neurons [28,29].

In boys, overweight status may be associated with earlier spermarche, while no association has been found between obesity and spermarche [21]. The association between BMI and puberty in boys remains controversial [30]. Some research has found a positive association between raised BMI and earlier pubertal development in boys [31]. However, other studies have concurred with these findings, noting that no advancement in pubertal development has been observed in obese boys [12]. A possible explanation was that adipose tissue possesses aromatase activity, which may increase the conversion of androgens to estrogens, leading to higher estrogen secretion in obese boys, thereby inhibiting the pubertal process [32–34]. However, this may not apply to boys who were overweight, as BMI is a benchmark indicator for overweight and obesity; overweight boys might not have a significant increase in adipose tissue but rather an increase in muscle mass. In addition, while estrogen stimulates leptin secretion, testosterone may suppress leptin secretion by adipocytes. The sexual dimorphism in leptin regulation during puberty could explain why obese girls tend to experience earlier pubertal development, while a similar pattern has not been observed in obese boys [35–37].

However, we found that the association between BMI and the age at menarche or spermarche was notably diminishing in both sexes. As the social economy develops, the influence of other elements on puberty, such as environmental endocrine disruptors, circadian rhythms, light pollution exposure, and so forth, is gaining importance [6,38]. Specifically, due to China's economic progress and increased quality of life, most children and adolescents could meet their daily nutritional needs. Moreover, children and adolescents' life patterns gradually change over time, including increased use of electronic devices, increased exposure to light pollution and environmental endocrine pollutants, sedentary behaviour, high psychological stress and circadian rhythm disorder, all of which may impact adolescent development [6,38,39]. Studies have shown that family structure and stressors are independently associated with early puberty [40,41]. Several studies have connected the presence of phthalates in toys, clothing, cosmetics, and building materials to an accelerated rate of breast development in both sexes [42,43]. It has also been demonstrated that the widely used chemical dichlorodiphenyltrichloroethane (DDT) is related to early menarche development [44,45]. Children who consume more milk and animal protein are more likely to experience early puberty [46,47]. Artificial light at night exposure may also influence the effects of melatonin and kisspeptin neurons on puberty.[39] Additionally, regarding biological mechanisms, it is hypothesised that with societal progression, the increased availability of high-calorie foods and a decline in physical activity levels have led to a gradual rise in overall human adiposity levels [48]. This may result in hormonal adaptations to accommodate the new normative levels of fat, thus altering the adipose or leptin threshold levels required to initiate of pubertal development. The effect of BMI on menarche or spermarche may be diminished by a rising number of additional factors. Future research should concentrate on other environmental impacts on the menarche or spermarche.

We also discovered that BMI had a stronger relationship with menarche or spermarche in rural areas, likely because the nutritional intake level of rural children and adolescents was lower than that of urban children in past decades, the proportion of thinness was larger in rural children and adolescents, and more affected by BMI. However, the urban-rural disparity was getting smaller over time. This may be a result of the rural child nutrition improvement program in China, where the prevalence of overweight and obesity among rural children and adolescents was on the rise [7]. Similar to the results of this study, the difference in overweight and obesity between urban and rural areas of Chinese children and adolescents also found a decreasing trend over time [49]. It is essential to promote a healthy lifestyle and counteract the prevalence of obesity, especially among rural children and adolescents [50,51].

In addition, our results provided some implications. We should still pay attention to children who are overweight and obese. Early menarche or spermarche may interact with overweight and obesity to worsen adult health. Moreover, future research should also explore the effects of other environmental pollutants on puberty development. We should provide the necessary health services, such as health education and health management, for children with early menarche or spermarche to promote their physical and mental health.

This is the first study to reveal a diminishing association between BMI and menarche or spermarche using continually comparable data from a large sample. However, the study had limitations. It cannot explore the causal relationship and the internal mechanism of BMI and puberty. In addition, BMI was not collected at the same time of menarche or spermarche onset but at the time of the interview. Recall bias might exist in the investigation. However, females have a superior memory of menarche, and research demonstrates that male self-reported spermarche was highly correlated with urine sperm [4]. Unknown confounding variables were not considered. The study exclusively investigated the association between BMI and pubertal development among children and adolescents within China, limiting the generalisability of the results. However, given China's rapid economic growth over recent decades, which may reflect other developing nations at similar stages, the findings could hold some relevance for these populations. Nevertheless, caution should be exercised when extrapolating these conclusions due to cultural, dietary, and health policy differences between countries.

Future research could employ prospective cohort designs to longitudinally track individual growth from childhood to adolescence longitudinally, thus enhancing understanding of the causal relationship between BMI and pubertal development. Additionally, applying causal inference statistical methods, such as instrumental variable analysis or regression discontinuity, could elucidate potential causal links between BMI and puberty. Furthermore, data collection at more precise and consistent time points regarding BMI and pubertal markers may reduce bias.

## CONCLUSIONS

In conclusion, there was a diminishing trend in the association between BMI and age at menarche or spermarche in both sexes. For girls, there was an apparent dose-response relationship; with the increase in BMI, menarche tended to be earlier. For boys, there might be a nonlinear association between BMI and spermarche. The results of urban-rural stratification showed a similar diminishing trend. Public health policymakers should consider these findings to optimise health promotion plans for children and adolescents, providing appropriate sexual health education and addressing obesity issues. In addition, future research should utilise longitudinal studies to explore the mechanisms between BMI and menarche or spermarche and identify other potential environmental factors that may affect puberty development.

## Additional material


Online Supplementary Document
