# Supplementary material for: Association between BMI and age at menarche or spermarche among both sexes: Findings from six successive national surveys in China
Source: J Glob Health. 2024 May 10;14:04099. doi: 10.7189/jogh.14.04099 (PMC11082623; doi:10.7189/jogh.14.04099)
Supplement: Online Supplementary Document [file jogh-14-04099-s001.pdf]

Table S1 Sample sizes by region (urban/rural) and sex (girl/boy) for children and adolescents participating in the CNSSCHs from 1995 to 2019

Age(y)	1995	2000	2005	2010	2014	2019
Urban Girls						
9	4125	4338	4632	3764	3765	3646
10	4402	4470	4909	4104	4002	3943
11	4408	4324	4834	4318	4214	4057
12	4396	4351	4779	4351	4349	4260
13	4393	4361	4905	4324	4414	4216
14	4381	4352	4854	4305	4443	4329
15	4393	4319	4929	4358	4446	4301
16	4395	4353	4915	4400	4466	4202
17	4402	4311	4838	4468	4471	4222
18	4365	3967	4349	4391	4268	3817
9-18	43660	43146	47944	42783	42838	40993
Rural Girls						
9	4061	4350	4702	3737	3806	3590
10	4354	4332	4652	3977	4078	4010
11	4243	4325	4763	4173	4223	4033
12	4199	4357	4676	4309	4343	4176
13	4159	4288	4788	4412	4409	4174
14	4194	4311	4750	4419	4426	4188
15	4190	4296	4879	4428	4475	4190
16	4121	4309	4814	4423	4462	4231
17	4128	4253	4848	4398	4476	4199
18	4090	4049	4634	4446	4253	3997
9-18	41739	42870	47506	42722	42951	40788

Urban Boys						
11	4393	4249	4242	3560	3499	3320
12	4393	4352	3887	3666	3660	3371
13	4392	4384	4033	3735	3804	3478
14	4402	4380	4204	3844	3917	3696
15	4403	4386	4495	4054	4070	3711
16	4341	4375	4585	4280	4315	3808
17	4394	4343	4572	4361	4327	3973
18	4333	4289	4555	4358	4151	3785
11-18	35051	34758	34573	31858	31743	29142
Rural Boys						
11	4346	4322	4188	3534	3552	3041
12	4327	4319	4176	3496	3720	3032
13	4345	4198	3895	3661	3900	3246
14	4339	4292	4052	3857	4024	3473
15	4338	4315	4502	3989	4205	3705
16	4338	4312	4582	4261	4288	3969
17	4334	4245	4615	4311	4380	3873
18	4318	4387	4713	4342	4222	3809
11-18	34685	34390	34723	31451	32291	28148

Table S2 Missing completely at random (MCAR) test of the sample

	Primary sample	Final sample	*P*-value
Age (Mean, SD)			
1995	14.0(2.7)	14.0(2.7)	0.931
2000	13.9(2.7)	13.9(2.7)	0.019
2005	14.0(2.7)	14.0(2.7)	0.783
2010	13.9(2.7)	14.1(2.7)	<0.001
2014	13.9(2.7)	14.1(2.7)	<0.001
2019	13.9(2.7)	14.0(2.7)	<0.001
Residence (N, %)			
1995			0.948
Urban	78742(50.7)	78711(50.7)	
Rural	76490(49.3)	76424(49.3)	
2000			0.595
Urban	81521(50.3)	77904(50.2)	
Rural	80542(49.7)	77260(49.8)	
2005			0.244
Urban	88545(50.3)	82517(50.1)	
Rural	87534(49.7)	82229(49.9)	
2010			0.277
Urban	80682(50.0)	74641(50.2)	
Rural	80805(50.0)	74173(49.8)	
2014			0.184
Urban	80356(50.0)	74581(49.8)	
Rural	80299(50.0)	75242(50.2)	

2019			0.244
Urban	79718(50.2)	70135(50.4)	
Rural	79029(49.8)	68936(49.6)	

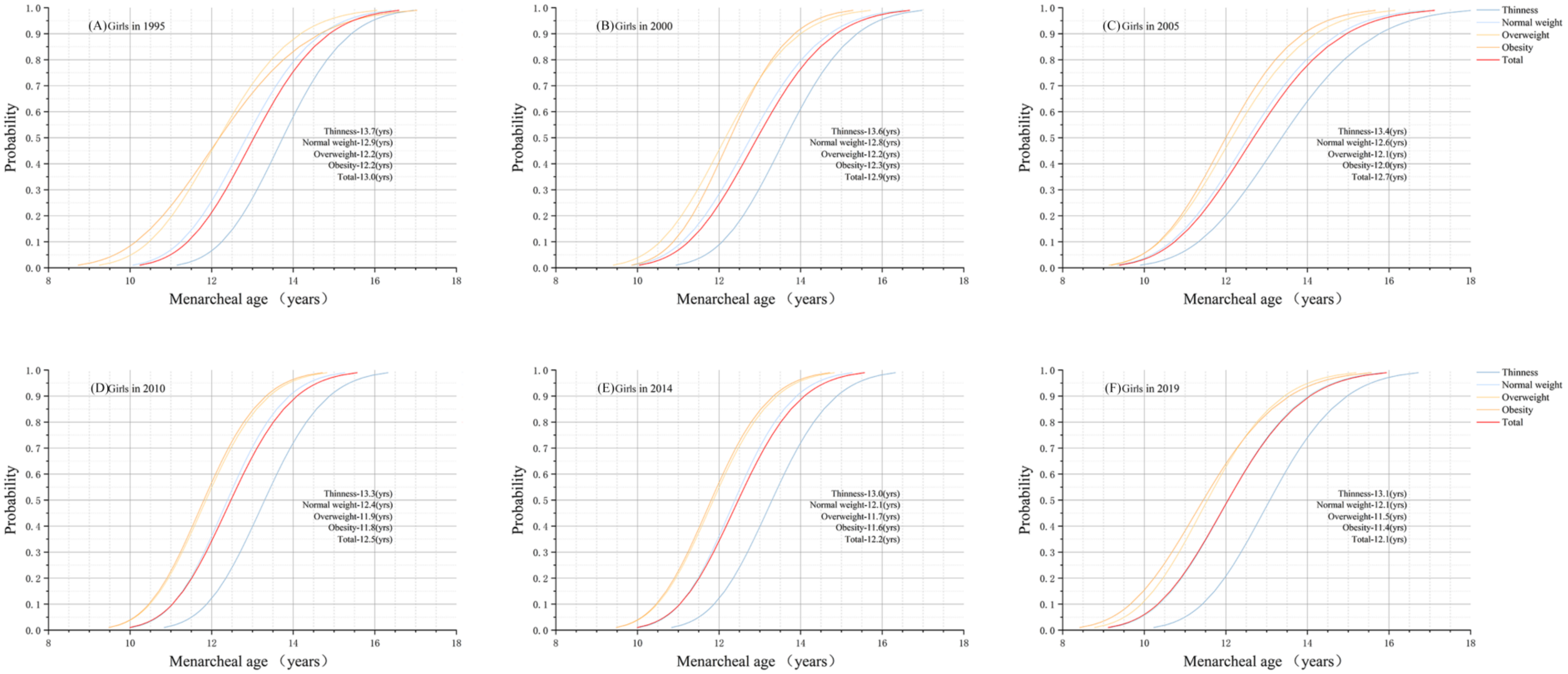

Figure S1 Probit plots of age at menarche by nutritional status from 1995 to 2019

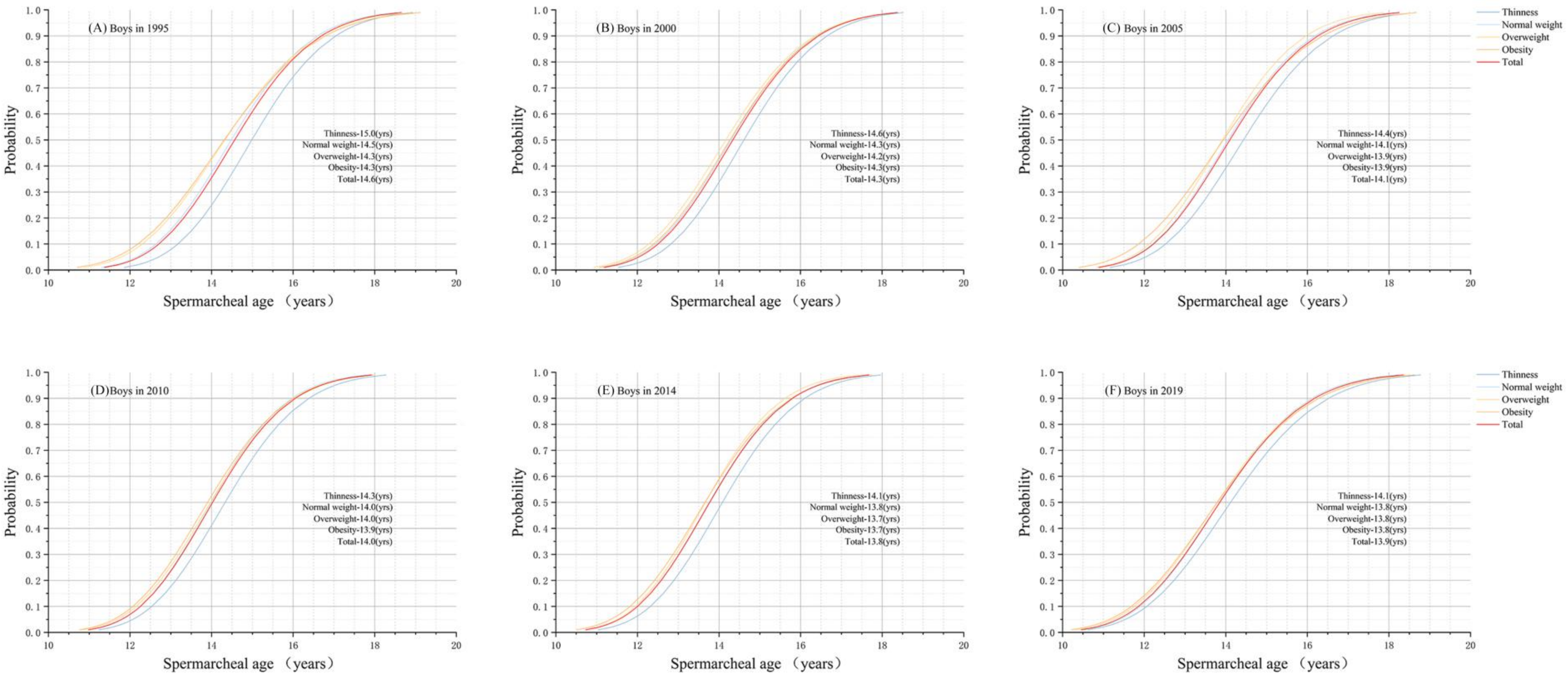

Figure S2 Probit plots of age at spermarche by nutritional status from 1995 to 2019

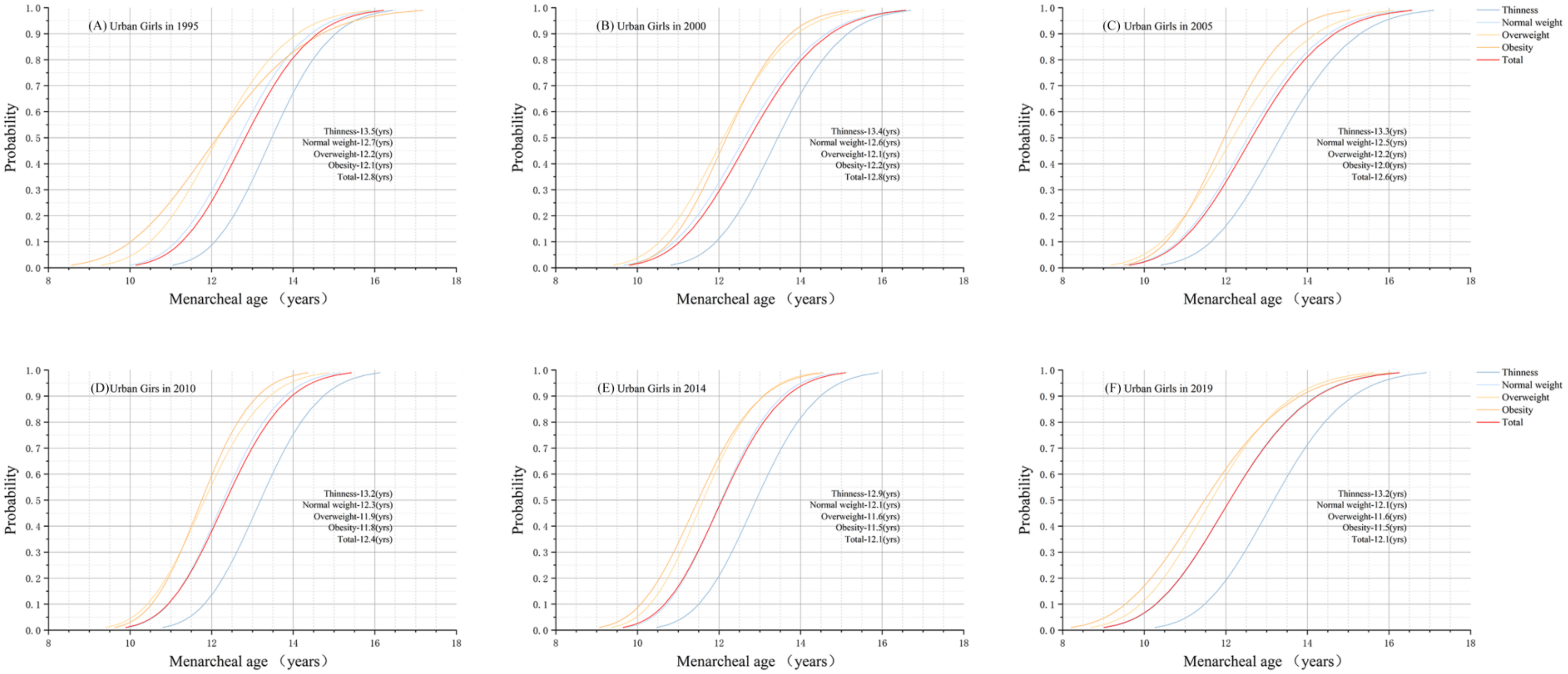

Figure S3 Probit plots of age at menarche by nutritional status in urban girls from 1995 to 2019

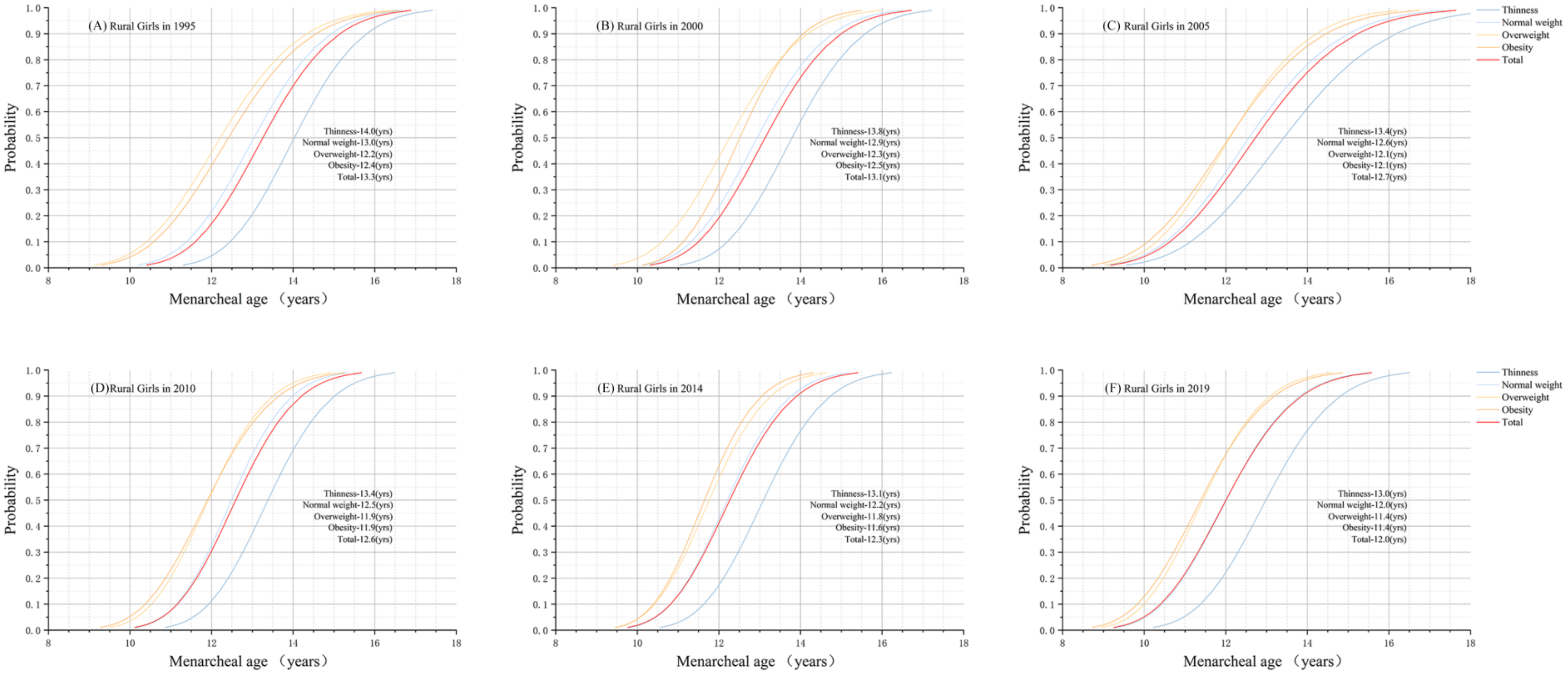

Figure S4 Probit plots of age at menarche by nutritional status in rural girls from 1995 to 2019

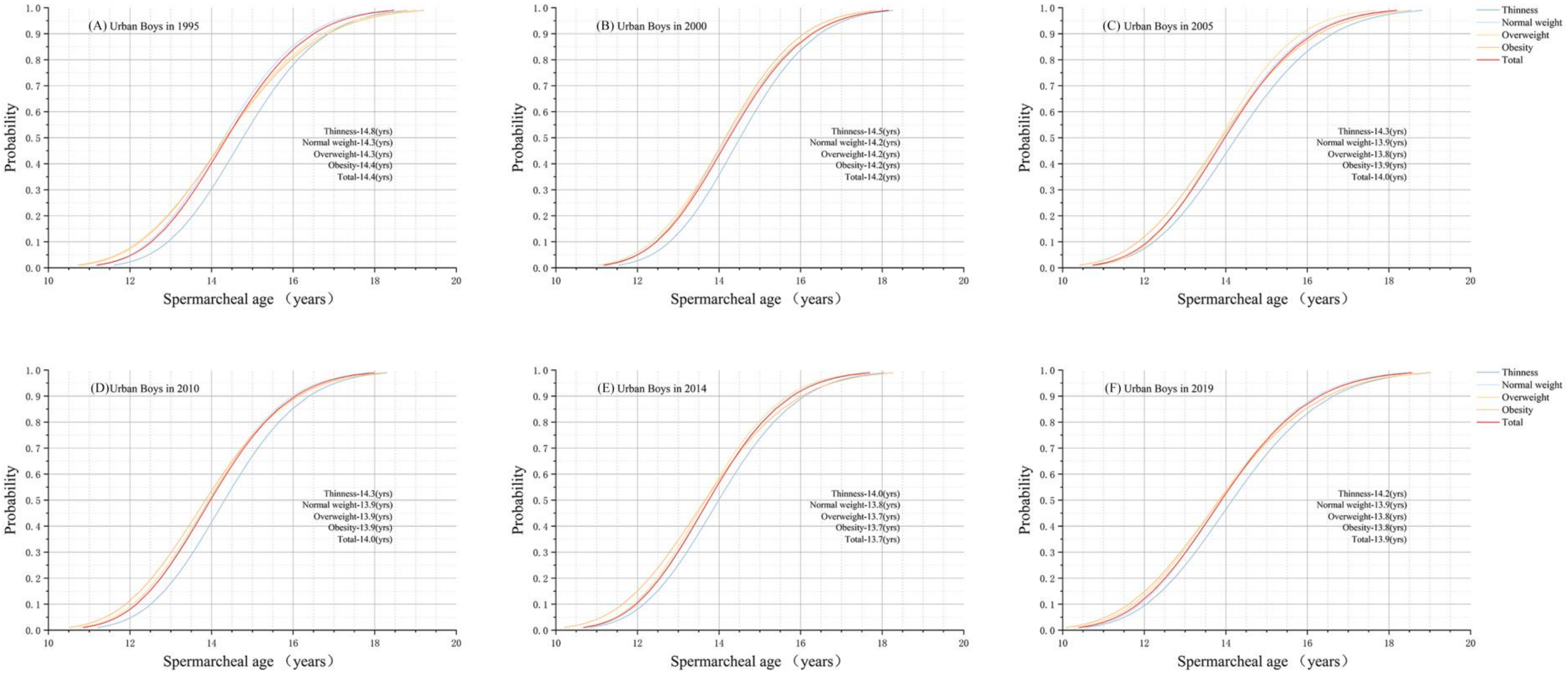

Figure S5 Probit plots of age at spermarche by nutritional status in urban boys from 1995 to 2019

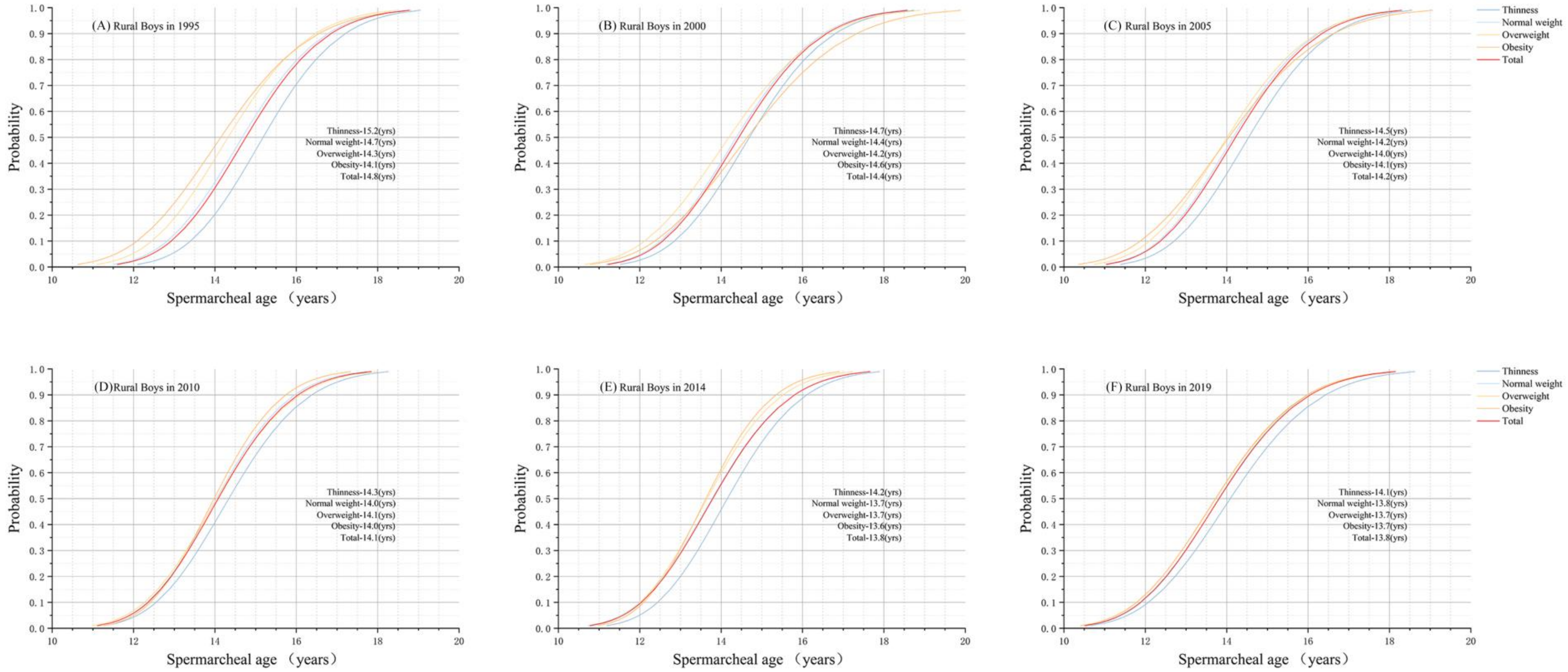

Figure S6 Probit plots of age at spermarche by nutritional status in rural boys from 1995 to 2019

	Girls OR (95%CI)	Girls PRSE	Girls Interaction *P* value	Boys OR (95%CI)	Boys PRSE	Boys Interaction *P* value
1995						
Thinness	0.23(0.21-0.25) ‡	3.5	.198	0.55(0.51-0.60) ‡	7.3	.889
Overweight	2.78(2.45-3.15) ‡	6.3	<.001	1.19(1.07-1.31) †	31.2	.001
Obesity	2.50(1.83-3.42) ‡	17.4	.162	1.22(0.99-1.39)	52.2	.065
P trend	< .001			< .001		
2000						
Thinness	0.26(0.24-0.28) ‡	3.6	.654	0.65(0.60-0.71) ‡	9.6	.620
Overweight	2.33(2.10-2.59) ‡	6.3	.017	1.20(1.10-1.30) ‡	24.6	.054
Obesity	2.12(1.69-2.66) ‡	15.4	.427	0.99(0.86-1.15)	125.0	.478
P trend	< .001			< .001		
2005						
Thinness	0.31(0.29-0.34) ‡	3.8	.073	0.68(0.63-0.74) ‡	10.8	.443
Overweight	1.68(1.55-1.81) ‡	7.7	.141	1.13(1.05-1.22) †	29.6	.324
Obesity	1.94(1.65-2.29) ‡	12.7	.214	1.14(1.01-1.28) †	46.1	.282
P trend	< .001			< .001		
2010						
Thinness	0.18(0.16-0.20) ‡	3.5	.130	0.63(0.56-0.70) ‡	11.5	.312
Overweight	2.54(2.33-2.77) ‡	4.8	.061	1.01(0.94-1.09)	316.7	.421
Obesity	2.67(2.22-3.20) ‡	9.5	.904	1.02(0.91-1.14)	316.7	.129
P trend	< .001			< .001		
2014						
Thinness	0.20(0.17-0.26) ‡	4.3	.774	0.63(0.56-0.71) ‡	13.4	<.001
Overweight	2.24(2.07-2.42) ‡	5.0	.832	1.13(1.06-1.21) ‡	27.4	<.001
Obesity	2.38(2.07-2.74) ‡	8.3	.663	1.07(0.98-1.17)	69.1	.165
P trend	< .001			< .001		
2019						
Thinness	0.22(0.19-0.25) ‡	4.9	.778	0.71(0.62-0.80) ‡	18.3	.314
Overweight	2.07(1.93-2.22) ‡	4.8	.087	1.10(1.03-1.18) †	34.0	.498
Obesity	2.25(2.01-2.53) ‡	7.3	.629	1.08(0.99-1.17)	55.4	.517
P trend	< .001			< .001		

Figure S7 Association between nutritional status and age at menarche or spermarche according to World Health Organization (WHO) references

Note: adjusted for age, province, socioeconomic status and residency status. ‡: $P<0.001$; †: $P<0.05$. OR: odds ratio; CI: confidence interval. BMI: body mass index. PRSE: percent relative standard error. Interaction *P* value: the interaction effect of residency status and nutritional status.

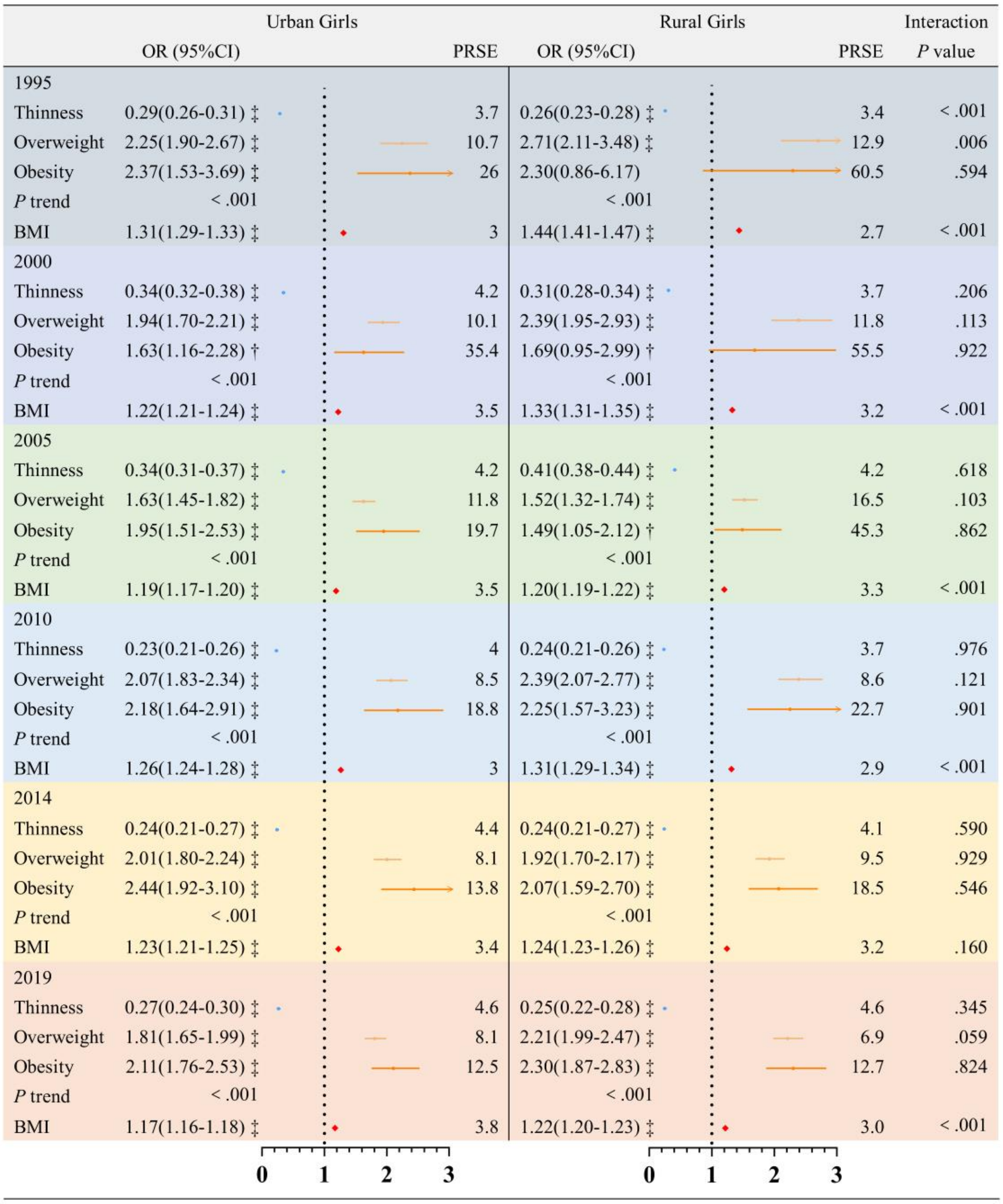

	Urban Girls OR (95%CI)	Urban Girls PRSE	Rural Girls OR (95%CI)	Rural Girls PRSE	Interaction *P* value
**1995**					
Thinness	0.29(0.26-0.31) ‡	3.7	0.26(0.23-0.28) ‡	3.4	< .001
Overweight	2.25(1.90-2.67) ‡	10.7	2.71(2.11-3.48) ‡	12.9	.006
Obesity	2.37(1.53-3.69) ‡	26	2.30(0.86-6.17)	60.5	.594
P trend	< .001		< .001		
BMI	1.31(1.29-1.33) ‡	3	1.44(1.41-1.47) ‡	2.7	< .001
**2000**					
Thinness	0.34(0.32-0.38) ‡	4.2	0.31(0.28-0.34) ‡	3.7	.206
Overweight	1.94(1.70-2.21) ‡	10.1	2.39(1.95-2.93) ‡	11.8	.113
Obesity	1.63(1.16-2.28) †	35.4	1.69(0.95-2.99) †	55.5	.922
P trend	< .001		< .001		
BMI	1.22(1.21-1.24) ‡	3.5	1.33(1.31-1.35) ‡	3.2	< .001
**2005**					
Thinness	0.34(0.31-0.37) ‡	4.2	0.41(0.38-0.44) ‡	4.2	.618
Overweight	1.63(1.45-1.82) ‡	11.8	1.52(1.32-1.74) ‡	16.5	.103
Obesity	1.95(1.51-2.53) ‡	19.7	1.49(1.05-2.12) †	45.3	.862
P trend	< .001		< .001		
BMI	1.19(1.17-1.20) ‡	3.5	1.20(1.19-1.22) ‡	3.3	< .001
**2010**					
Thinness	0.23(0.21-0.26) ‡	4	0.24(0.21-0.26) ‡	3.7	.976
Overweight	2.07(1.83-2.34) ‡	8.5	2.39(2.07-2.77) ‡	8.6	.121
Obesity	2.18(1.64-2.91) ‡	18.8	2.25(1.57-3.23) ‡	22.7	.901
P trend	< .001		< .001		
BMI	1.26(1.24-1.28) ‡	3	1.31(1.29-1.34) ‡	2.9	< .001
**2014**					
Thinness	0.24(0.21-0.27) ‡	4.4	0.24(0.21-0.27) ‡	4.1	.590
Overweight	2.01(1.80-2.24) ‡	8.1	1.92(1.70-2.17) ‡	9.5	.929
Obesity	2.44(1.92-3.10) ‡	13.8	2.07(1.59-2.70) ‡	18.5	.546
P trend	< .001		< .001		
BMI	1.23(1.21-1.25) ‡	3.4	1.24(1.23-1.26) ‡	3.2	.160
**2019**					
Thinness	0.27(0.24-0.30) ‡	4.6	0.25(0.22-0.28) ‡	4.6	.345
Overweight	1.81(1.65-1.99) ‡	8.1	2.21(1.99-2.47) ‡	6.9	.059
Obesity	2.11(1.76-2.53) ‡	12.5	2.30(1.87-2.83) ‡	12.7	.824
P trend	< .001		< .001		
BMI	1.17(1.16-1.18) ‡	3.8	1.22(1.20-1.23) ‡	3.0	< .001

Figure S8 Association between BMI and menarche stratified by residency status from 1995 to 2019

Note: adjusted for age, province and socioeconomic status. ‡: P<0.001; †: P<0.05. OR: odds ratio; CI: confidence interval. BMI: body mass index. PRSE: percent relative standard error. Interaction P value: the interaction effect of residency status and BMI/nutritional status.

	Urban boys		Rural boys		Interaction
	OR (95%CI)	PRSE	OR (95%CI)	PRSE	*P* value
1995					
Thinness	0.56(0.51-0.61) ‡	7.8	0.55(0.50-0.59) ‡	7.1	.295
Overweight	1.01(0.89-1.15)	590.9	1.45(1.16-1.81) †	30.5	< .001
Obesity	1.00(0.73-1.35)	314.0	1.71(0.94-3.11)	56.8	.029
P trend	< .001		< .001		
BMI	1.07(1.06-1.08) ‡	9.1	1.16(1.14-1.18) ‡	5.4	< .001
2000					
Thinness	0.72(0.66-0.79) ‡	14.3	0.68(0.63-0.74) ‡	10.6	.420
Overweight	1.03(0.93-1.15)	183.3	1.26(1.06-1.49) †	37.3	.062
Obesity	1.02(0.82-1.26)	605.6	0.76(0.52-1.12)	73.2	.157
P trend	< .001		< .001		
BMI	1.04(1.03-1.05) ‡	12.8	1.08(1.06-1.09) ‡	9.7	.042
2005					
Thinness	0.69(0.62-0.76) ‡	13.1	0.62(0.57-0.68) ‡	9.1	.400
Overweight	1.05(0.95-1.15)	108.9	1.21(1.05-1.39) †	37.6	.373
Obesity	1.00(0.83-1.20)	470.0	1.21(0.90-1.63)	78.6	.555
P trend	< .001		< .001		
BMI	1.04(1.03-1.05) ‡	14.7	1.08(1.07-1.09) ‡	8.0	< .001
2010					
Thinness	0.66(0.59-0.74) ‡	14.0	0.70(0.63-0.77) ‡	15.0	.254
Overweight	0.99(0.90-1.09)	612.5	0.93(0.82-1.05)	80.8	.261
Obesity	1.03(0.87-1.21)	330.8	0.93(0.73-1.19)	182.6	.400
P trend	< .001		< .001		
BMI	1.02(1.01-1.03) ‡	20.8	1.03(1.02-1.05) ‡	18.2	< .001
2014					
Thinness	0.74(0.65-0.84) ‡	20.9	0.65(0.58-0.73) ‡	13.5	< .001
Overweight	1.09(1.00-1.20)	51.7	1.12(1.01-1.24) †	47.3	.006
Obesity	1.06(0.91-1.23)	135.7	1.09(0.91-1.32)	106.7	.238
P trend	< .001		< .001		
BMI	1.02(1.01-1.03) ‡	21.7	1.04(1.03-1.05) ‡	12.8	.160
2019					
Thinness	0.71(0.63-0.81) ‡	19.5	0.71(0.63-0.81) ‡	18.6	.940
Overweight	1.08(0.99-1.18)	57.1	1.08(0.98-1.18)	68.1	.981
Obesity	1.09(0.96-1.24)	78.3	1.08(0.93-1.25)	94.9	.726
P trend	< .001		< .001		
BMI	1.02(1.01-1.03) ‡	19.0	1.02(1.02-1.03) ‡	20.8	< .001

Figure S9 Association between BMI and spermarche stratified by residency status from 1995 to 2019

Note: adjusted for age, province and socioeconomic status. ‡: *P*<0.001; †: *P*<0.05. OR: odds ratio; CI: confidence interval. BMI: body mass index. PRSE: percent relative standard error. Interaction *P* value: the interaction effect of residency status and BMI/nutritional status.
